# Breaking the Chain: Strategies to Stem Adenovirus Spread in Pakistan

**DOI:** 10.1111/irv.13287

**Published:** 2024-04-08

**Authors:** Rayyan Vaid, Afra Sohail, Raam Kumar, Areeba Fareed

**Affiliations:** ^1^ Department of Medicine Karachi Medical and Dental College Karachi Pakistan; ^2^ Department of Medicine Jinnah Sindh Medical University Karachi Pakistan

**Keywords:** adenovirus, Pakistan, viral infections

## Abstract

Adenovirus, a common respiratory pathogen, has witnessed a notable rise in incidence rates across various regions in Pakistan. Utilizing epidemiological data and climate records, this research discerns a potential linkage between the burgeoning adenovirus cases and alterations in regional climate patterns. Through statistical analysis and modeling techniques, the study aims to elucidate the relationship between climatic variables, such as temperature, humidity, and precipitation, and the prevalence of adenovirus infections. Understanding these dynamics is crucial for developing effective public health interventions and preparedness strategies to mitigate the impact of adenovirus outbreaks in Pakistan. Furthermore, this research contributes to the broader discourse on the intersection of infectious diseases and climate change, highlighting the need for comprehensive adaptive measures to address emerging health challenges in a changing environment.

Dear Editor,

Adenovirus is on the rise in Pakistan, owing to changes in the climate and environmental pollution [1, 2]. Adenovirus, a double‐stranded DNA genome non‐enveloped virus, is known for infecting the respiratory and gastrointestinal tracts, as well as causing conjunctivitis. Given its wide‐ranging symptoms, including cough, diarrhea, and eye redness, adenovirus poses a significant health threat, especially to children who are more susceptible due to frequent face‐ and mouth‐touching habits [3]. Rarely deaths are reported from the infection of the virus; however, adenovirus‐related pneumonia may cause death among children [4]. Pakistan is among the countries with a significantly high prevalence of adenovirus [5].

The frequency of adenovirus is increasing significantly, causing concern. According to current data from Karachi's Dr. Ruth KM Pfau Civil Hospital in February 2024, in just 2 days, 782 and 1275 cases of adenovirus causing severe respiratory tract infection had been reported [6]. This increase is in line with a pattern that was noticed in February 2023, when over 1000 people with symptoms including headaches, runny noses, fever, and chills visited government‐run hospitals per day [1].

Furthermore, an outbreak of adenoviral conjunctivitis in September 2023, affected a significant portion of the population, with Punjab bearing the brunt. The outbreak led to an alarming 86,133 cases, with an average of 13,000 new cases reported daily, highlighting the urgent need for containment measures [5]. Moreover, during the same period, Nishtar Hospital of Multan also reported about 100 cases in a single day [7]. This accentuates the urgent need for proactive measures to prevent future adenovirus outbreaks.

Climate change significantly influences adenovirus outbreaks, with research indicating peak occurrences during the winter and spring seasons [2, 8, 9]. This is the primary reason of the spread of adenovirus in Pakistan during these days. Several other factors include poor hygiene practices, airborne droplets, fecal contamination, and limited access to clean water and sanitation infrastructure. Additionally, the impact of these changes are exacerbated by the changes in climate [1, 2, 3].

While Pakistan allocates only $4.2 per person for medical expenses, the WHO recommends a $34 per capita health fund. This significant disparity creates challenges during medical emergencies such as outbreaks of adenoviral conjunctivitis. Government financial support of the health sector and cheap treatment and immunization programs are essential for effectively combating future epidemics [5]. To reduce the spread of adenoviruses and prevent further outbreaks, stronger sanitary practices, public awareness efforts, and medical treatments are essential. Building infrastructure and capability in the healthcare sector is necessary for effective outbreak management. Measures among individuals should be taken such as washing hands, avoiding close contact, proper disposal of waste, and isolation of infected, which can play a huge role in mitigating the transmission of this virus and in reducing the burden of outbreaks effectively.

In conclusion, the recent surge in adenovirus cases in Pakistan necessitates a multifaceted approach that includes both preventive measures and effective treatment strategies. By implementing comprehensive public health interventions, bolstering healthcare infrastructure, and investing in research and development, we can lessen the impact of adenovirus outbreaks and safeguard the well‐being of our communities. Government agencies, healthcare providers, and the general public must work together to address this pressing public health issue and ensure a healthier future for all.

## Author Contributions

**Rayyan Vaid:** Conceptualization; Writing – review and editing; Writing – original draft; Supervision; Project administration. **Afra Sohail:** Writing – original draft; Writing – review and editing. **Raam Kumar:** Writing – original draft; Writing – review and editing. **Areeba Fareed:** Writing – review and editing.

## Conflicts of Interest

The authors declare no conflicts of interest.

### Peer Review

The peer review history for this article is available at https://www.webofscience.com/api/gateway/wos/peer‐review/10.1111/irv.13287.
